# An Improved Bionic Artificial Lemming Algorithm for Global Optimization and Cloud Task-Scheduling Problems

**DOI:** 10.3390/biomimetics10090572

**Published:** 2025-08-28

**Authors:** Yuyong Tan, Jianfeng Wang, Bin Wang

**Affiliations:** 1 School of Mathematics and Computing Sciences, Guilin University of Electronic Technology, Guilin 541004, China; 2 South Korea College of Design, Hanyang University, Ansan 15588, Gyeonggi-do, Republic of Korea; wangbin19045@hanyang.ac.kr

**Keywords:** intelligent optimization algorithms, artificial lemming algorithm, cloud scheduling, slow convergence speed

## Abstract

The intelligent optimization algorithm has become a key tool in complex and intertwined engineering and science fields. However, with the increasing complexity of the problem and the rapid expansion of the data scale, the performance of the algorithm has been challenged unprecedentedly. The artificial lemming algorithm has gradually emerged because of its unique structural design and efficient optimization performance and has been widely recognized by academic circles. However, in the face of more complex and challenging optimization and scheduling problems, it also exposed some obvious shortcomings. For example, the dispersion of the initial individual set in the algorithm is low, which leads to the low accuracy of the optimal solution. In addition, the exploitation ability of the algorithm is relatively weak, which leads to a slow convergence speed. Fortunately, this paper proposes an improved artificial lemming algorithm. Based on the in-depth analysis of the original algorithm, aiming at addressing the shortcomings of the original algorithm, some innovative mechanisms are introduced. In order to verify the effectiveness of the improved algorithm, a large number of experiments are carried out through global optimization test problems. The experimental results show that the performance of the algorithm has been obviously improved, and the accuracy and convergence speed of the solution are obviously better than the original algorithm and some baseline algorithms. In addition, this paper also applies the improved artificial travel algorithm to the cloud scheduling problem. These experimental results further verify the feasibility and effectiveness of this method in practical application and provide strong support for its application in a wider range of fields.

## 1. Introduction

The widespread adoption of cloud computing has promoted the development of various fields. Diseases can be detected and identified through cloud databases [1], the distribution of crops can be forecast through cloud technology [2], and species of crops can be modeled and identified [3,4,5]. Based on cloud technology, factors such as compounds can be quickly predicted [6,7,8], and the cross-collaboration of multiple technologies can be realized [9,10,11,12]. Cloud technology is displayed in all aspects. However, with the surge of data flow, the complexity of the problem also shows a rapid upward trend. Therefore, researchers address this problem through the use of algorithms. In the development wave of modern intelligent algorithms, the design and iteration of a large number of optimization algorithms have deeply integrated the core knowledge of bionics. These algorithms meticulously simulate the unique structures, efficient functions, and adaptive behaviors that organisms have developed over billions of years of evolution in nature. For instance, the cooperative foraging patterns of ant colonies, the group migration mechanisms of birds, and the mutation and selection rules of genes, etc., are used to construct their own optimization logic and operational framework.

In the field of food content detection, Felix Y.H. Kutsanedzie et al. proposed the use of Fourier transform near-infrared spectroscopy (FT-NIRS) in conjunction with a stoichiometric algorithm and Fourier transform infrared spectroscopy, which can effectively detect fungal content [13]. Zhiming Guo et al. put forward Synergy Interval Partial Least Squares Ant Colony Optimization (SIPLS-ACO) by combining a regression model, an ant colony optimization algorithm, and other strategies. Experiments show that this method can effectively evaluate the content of zearalenone [14]. Qin Ouyang et al. combined a stoichiometric algorithm with a portable electronic tongue to effectively detect the total content of theaflavins in black tea [15]. Hui Jiang et al. used a genetic algorithm, ant colony optimization, and particle swarm optimization to optimize the sensor and combined with neural network and other tools to propose an effective method to detect the content of fatty acids in flour [16]. Xin Zhou et al. proposed a novel detection algorithm of metal content in food in cooperation with Stack Convolution Auto Encoder (SCAE) and a deep learning algorithm [17]. Additionally, some algorithms are applied to the detection of germs in food [18,19,20,21,22]. In the field of crop classification, Yating Li et al. combined the Variable Iterative Space Shrinkage Approach (VISSA) and Model Adaptive Space Shrinkage (MASS) algorithms to propose an efficient classification algorithm for tea varieties [23]. Xin Zhou et al. proposed the data-level information fusion method coupled with a Variable Iterative Space Shrinkage Approach combined with the Grasshopper Optimization Algorithm Support Vector Machine (VISSA-GOA-SVM) algorithm, which can effectively identify the residues of different heavy metals in lettuce [24]. Ernest Bonah et al. combined a particle swarm optimization algorithm and a support vector machine to propose an efficient bacterial species identification algorithm [25]. Ningqiu Tang et al. proposed a method for detecting the species of wolfberry based on hyperspectral imaging technology [26] and some other food detection and classification algorithms [27,28,29]. In terms of index detection, Muhammad Arslan et al. proposed an improved near-infrared method for the efficient quantitative detection of antioxidant activity in BW samples [30]. Jingjing Wang et al. proposed a fast and efficient algorithm for detecting the types of matcha samples [31]. Jun Sun et al. optimized the parameters in the support vector regression model using the gray wolf optimization algorithm and proposed a high-precision moisture content detection method [32]. Moreover, some algorithms are applied to detection platforms or systems [33,34,35,36].

Among numerous algorithms, the artificial lemming algorithm (ALA) has attracted extensive attention from researchers due to its simple structure and strong practicality [37]. The ALA takes the unique survival and migration behaviors of lemmings in nature as its inspiration and achieves efficient solutions to complex optimization problems by precisely simulating their biological characteristics and group dynamics. However, it still has obvious drawbacks when dealing with complex-scale problems, such as insufficient initial population richness and low convergence precision of the optimal value. Fortunately, the improved artificial lemming algorithm approach proposed herein can not only effectively solve global optimization problems, but also be applied to cloud task-scheduling scenarios. The main contributions of this article are as follows:

(1) This paper proposes a collaborative second-order Bernstein polynomial and chaotic mapping function initialization strategy (BPSC). By perturbing individual positions in the initial stage of the algorithm, the population dispersion is enhanced, thereby increasing the closeness of the algorithm’s solution to the optimal value.

(2) By introducing a quadratic interpolation random mutation strategy during the exploitation, the mutation rate is effectively controlled to prevent individuals from escaping the current optimal trap, further enhancing the algorithm development capability.

(3) In the exploration stage of the algorithm, an adaptive evolutionary strategy is introduced. This is because premature convergence will easily lead to the algorithm falling into the local optimal trap. Through the adaptive exploration mechanism of the unknown solution space, the population richness of the algorithm in the exploration stage can be improved. Then the premature convergence of the algorithm can be effectively avoided.

(4) Through the collaborative implementation of the above strategies, an improved artificial lemming algorithm is proposed. It was applied to the CEC2021 problem and the cloud task-scheduling problem, demonstrating that it is a highly robust algorithm.

The outline of the remaining part of the article is as follows: Section 2 conducts relevant work on the current background, Section 3 describes the initial artificial lemming algorithm, Section 4 proposes an improved artificial lemming algorithm, Section 5 analyzes the performance of the proposed algorithm through experiments, and Section 6 discusses the research conclusions and future development.

## 2. The Related Work

In this section, the advantages and disadvantages of some current improved algorithms are mainly analyzed, as well as the strengths and weaknesses of some task-scheduling algorithms or models.

### 2.1. A Review of Improved Algorithms

Yan et al. proposed an improved deep learning algorithm for multimodal arc detection networks [38]. The experimental results show that this method achieves excellent detection performance with the minimum amount of data in the complex railway environment, especially having significant application potential in railway system condition monitoring and anomaly detection. Xin Wang et al. proposed an adaptive fusion domain cyclic variational generative adversarial network [39]. Songguang Zhao et al. developed an online visible/near-IR spectroscopy detection system for the surveillance of the concentrations of soluble sugar, total acid, and bacteria. By combining the Sparrow Search Algorithm (SSA) [40] with the Whale Optimization Algorithm (WOA) [41] and the African Vulture Optimization Algorithm (AVOA) [42], the framework’s precision was advanced. However, since the parameters of the optimization algorithm cannot be dynamically adjusted, to a certain extent, this method still has some room for improvement in terms of the accuracy of the detection model. Hui Jiang et al. utilized the particle swarm optimization algorithm to optimize the color components of different color-sensitive points [43], and in collaboration with the backpropagation neural network, innovatively proposed a sensory evaluation method for the aroma quality of black tea based on the olfactory and visual sensing system. However, the computational cost of neural network architectures is high, and they are sensitive to initial parameters. Therefore, if the initial value is too large, then it may cause the activation function to enter the saturation zone, the gradient to approach 0, and the parameters cannot be updated. Yuhan Ding et al. adopted the corpuscle flock optimization algorithm to perfect the variables in the sustain vector mechanism and put forward a tea quality rank categorization approach based on near-infrared spectrography [44], which significantly improved the classification accuracy of tea categories. However, when the data scale is large, the training speed is slow, which in turn leads to a longer system response time.

### 2.2. Overview of Task Scheduling

Chenbo Ding et al. proposed a blockchain-based wide-area agricultural machinery resource-scheduling system and agricultural machinery-scheduling optimization model based on the business models of different regions [45], effectively reducing the production costs of wide-area agriculture and improving the utilization rate of agricultural machinery resources. However, the resource cost is not considered evenly in this model. Yiyuan Pang et al. artificially reduced irrigation energy consumption by introducing the branch pipe allocation queue and the number of branch pipe allocations [46] and proposed an efficient irrigation resource-scheduling system. Optimizing the branch pipe allocation queue and the number of allocations can achieve an 8% energy savings. However, the optimization algorithm is based on certain assumptions and simplified model establishment. Thus, there are problems such as a stable water flow state in the pipeline and ignoring local resistance. Fatma A. Omara et al. proposed an improved genetic algorithm [47], which effectively solves the scheduling and mapping problems from priority-constrained task graphs to processors. However, in the model, the cost of resource bandwidth is not taken into account. Thus, the model has certain flaws. Xianyong Wei proposed an improved ant colony optimization algorithm [48], which effectively solves problems such as unbalanced load and low utilization rate of system resources. However, in this model, the cost of task execution has not been taken into account. Therefore, in real life, this model still needs improvement. Suji Zhu et al. proposed the Chaos-Cauchy Fireworks Algorithm (CCFWA) for the multi-limitation problem of multi-machine collaborative operation in machinery [49]. This method can effectively allocate agricultural resources.

## 3. Introduction to Optimization Problem

This section will present a systematic and exhaustive description of the optimization problems examined in this paper. Specifically, it will first provide an in-depth introduction to CEC2021, covering essential information such as problem names, optimal values, and dimensions, thereby laying the groundwork for subsequent optimization analyses. Furthermore, the optimization objectives of the cloud task-scheduling problem will be elaborated in detail, and the evaluation metrics used to assess the quality of scheduling solutions will also be explained so as to offer a comprehensive overview of the optimization problems involved in this test.

### 3.1. Introduction to Global Optimization Problem

The CEC2021 benchmark was released by the IEEE Computational Intelligence Society in November 2020. Rather than being designed from scratch, the test suite recombines ten classical functions carefully selected from the single-objective, bound-constrained numerical optimization benchmarks of CEC2014 and CEC2017, preserving the original rotation, shifting, and composition transformations. Consequently, CEC2021 integrates the strengths and mitigates the weaknesses of its two predecessors, notably eliminating the “center bias” weakness that arose in CEC2014/2017 when the global optimum lay at the origin or the center of the feasible region. The resulting benchmark offers researchers from 2021 onward a lightweight, easily reproducible, and controllably challenging unified platform for rapid performance comparisons between new and existing algorithms. Its detailed composition is presented in Table 1. In Table 1, F1 corresponds to a unimodal function—i.e., a problem possessing a single global optimum. F2–F4 represent basic functions. F5–F7 are hybrid functions, each composed of several distinct problem types, whereas F8–F10 are composition functions formed by combining multiple problems.

### 3.2. Introduction to Cloud Task-Scheduling Problem

In cloud task scheduling, the relationship between tasks and computing resources can be represented by a binary matrix. If there are M cloud tasks and K computing resources, then this binary matrix can be represented by U.

(1) $$U={\left[\begin{array}{l} u_{11\;}\;\ldots \;u_{1j\;}\;\ldots \;u_{1K}\\ \;\vdots \;\;\;\;\vdots \;\;\;\;\vdots \\ u_{i1\;\;}\;\ldots \;u_{ij\;\;}\;\ldots \;u_{iK}\\ \;\vdots \;\;\;\;\vdots \;\;\;\;\vdots \\ u_{M1}\;\ldots \;u_{Mj}\;\ldots \;u_{MK} \end{array}\right]}_{M\cdot K}$$

Here, $u_{ij}=1$ indicates that the ith task is being calculated on the jth computing resource. Since each task can only be computed on one computing resource at a time, the total sum of each row is 1.

In cloud task scheduling, the time cost is a critical evaluation metric; it is generally linked to both task parameters and the processing capability of virtual machines. In this paper, the time cost is defined as the ratio of the computational resources required by a task to the computational resources provided by the assigned VM, as expressed in Equation (2).

(2) $$TC=\sum_{i=1}^{M}\sum_{j=1}^{K}u_{ij}\cdot \frac{Ca_{ti}}{Ca_{vj}}$$

Here, $Ca_{ti}$ represents the computing resources required for the ith task, and $Ca_{vj}$ represents the computing resource capacity of the jth virtual machine. Therefore, our objective is to minimize the overall system time, i.e., $\min\left\{TC\right\}$.

Beyond time cost, the system’s load cost is another critical metric. An unbalanced load can lead to low resource utilization and may also overload high-performance virtual machines, thereby shortening their lifespan. Here, the load cost is defined as the ratio of the load capacity required by a task to the load capacity available in the assigned computing resource, as given in Equation (3).

(3) $$LC=\sum_{i=1}^{M}\sum_{j=1}^{K}u_{ij}\cdot \frac{Lo_{ti}}{Lo_{vj}}$$

here, $Lo_{ti}$ represents the load resource required for the ith task, and $Lo_{vj}$ represents the load resource capacity of the jth virtual machine. Therefore, our objective is to minimize the overall load cost, i.e., $\min\left\{LC\right\}$.

In all task-scheduling scenarios, monetary cost is often the metric most valued by service consumers. This cost depends not only on the computational power required by the task but also on the bandwidth resources it consumes. In this paper, the price cost is defined by Equation (4).

(4) $$PC=\sum_{i=1}^{M}\sum_{j=1}^{K}u_{ij}\cdot \frac{unt\cdot Ca_{ti}\cdot RB_{ti}}{Ca_{vj}\cdot RB_{vj}}$$

Here, $RB_{ti}$ represents the resource throughput needed for the ith task, and $RB_{vj}$ represents the resource capacity demanded of the jth virtual machine. unt is a price-related parameter, and unt=5 is specified. Therefore, our objective is to minimize the overall price cost, i.e., $\min\left\{PC\right\}$.

Therefore, the total price cost is expressed by Equation (5).

(5) $$ToC=\min\left\{\beta 1\cdot TC+\beta 2\cdot LC+\beta 3\cdot PC\right\}$$

Here, β1, β2, and β3 denote the relative weights assigned to the optimization of time cost, load cost, and price cost, respectively. Since this experiment imposes no special preference on any single aspect, all three weights are set to one-third. Because the units of time, load, and price costs differ, Equation (5) alone might yield an inadequate computation. Consequently, a min–max normalization is applied to the three costs, bringing them to the same order of magnitude; the normalized cost computation is expressed in Equation (6).

(6) $$\left\{\begin{array}{l} TolTC=\frac{1}{M}\cdot \sum_{i=1}^{M}\sum_{j=1}^{K}u_{ij}\cdot \frac{Ca_{ti}/Ca_{vj}}{\underset{\forall i,j}{\max}\left\{Ca_{ti}/Ca_{vj}\right\}}\\ TolLC=\frac{1}{M}\cdot \sum_{i=1}^{M}\sum_{j=1}^{K}u_{ij}\cdot \frac{Lo_{ti}/Lo_{vj}}{\underset{\forall i,j}{\max}\left\{Lo_{ti}/Lo_{vj}\right\}}\\ TolPC=\frac{1}{M}\cdot \sum_{i=1}^{M}\sum_{j=1}^{K}u_{ij}\cdot \frac{\left(unt\cdot Ca_{ti}\cdot RB_{ti}\right)/\left(Ca_{vj}\cdot RB_{vj}\right)}{\underset{\forall i,j}{\max}\left\{\left(unt\cdot Ca_{ti}\cdot RB_{ti}\right)/\left(Ca_{vj}\cdot RB_{vj}\right)\right\}} \end{array}\right.$$

Therefore, the ultimate optimization objective for the cloud task-scheduling is:

(7) $$TolC=\min\left\{\frac{1}{3}\cdot TC+\frac{1}{3}\cdot LC+\frac{1}{3}\cdot PC\right\}$$

## 4. Standard Artificial Lemming Algorithm

The ALA mainly simulates four behaviors of lemmings, including long-distance migration, lemming burrowing, food search, and evading enemies. By analyzing the four behaviors, the individuals in its population are brought closer to the optimal ones.

### 4.1. Principle of Algorithm

Within the primary group of the standard artificial lemming algorithm, the randomness of individual positions is relatively high, which is specifically determined by the upper and lower bounds of the problem. The specific process is shown in Equation (8).

(8) $$Po_{i}=\mathrm{low}+\mathrm{rand}\cdot \left(\mathrm{upp}-\mathrm{low}\right)$$

Here, $Po_{i}$ is the i individual amid the original group, low represents the lower space boundary of the problem, upp is the upper space boundary of the problem, and rand is a randomly generated random number with a value between 0 and 1. rand satisfies a continuous and uniform distribution U(0,1), and the generated interval is strictly an open interval (0,1). Theoretically, 0 or 1 will never occur. rand presents a perfect continuous and uniform distribution U(0,1), without observable systematic deviations or periodic fringes.

Based on this, the ALA will dynamically switch to the exploration or development stage according to the specific value of the selection factor: When the selection factor is within a specific range, the algorithm will focus on the implementation of the exploration process, and through extensive search in the solution space, potential high-quality regions will be mined. When the selection factor is in another range, it will turn to the development process and conduct an in-depth and detailed search of the better areas found. The selection factor is calculated by Equation (9).

(9) $$SF=4\cdot \arctan\left(1-\frac{loop}{Maxloop}\right)\cdot ln\left(\frac{1}{uik}\right)$$

Here, $arctan\left(\cdot \right)$ represents the arctangent function, $ln\left(\cdot \right)$ represents a logarithmic function with base e, and uik represents a random number between 0 to 1. uik and rand have the same distribution pattern. loop represents the current number of iterations and Maxloop expresses the maximum number of iterations.

Then, based on the calculated value of the selection factor, the corresponding stage is executed. How to simulate the behavior of lemmings at each stage is determined by random numbers. If SF>1, then the lemming information is updated during the exploration phase, which is specifically manifested as Equation (10).

(10) $$Po_{inew}=\left\{\begin{array}{cc} Po_{\mathrm{best}}+F\cdot \delta \cdot \left(\left(\varepsilon \cdot \left(Po_{\mathrm{best}}-Po_{i}\right)\right)+\left(1-\varepsilon \right)\cdot \left(Po_{i}-Po_{RN}\right)\right) & \;\mathrm{if}\;\eta <0.3\\ Po_{i}+F\cdot \rho \cdot \left(Po_{\mathrm{best}}-Po_{\varphi }\right) & \;\mathrm{otherwise} \end{array}\right.$$

Here, η is a random variable with values ranging from 0 to 1, and the distribution patterns are all consistent with uik. $Po_{\mathrm{best}}$ represents the current optimal individual in the population, and δ is used to generate random vectors that follow a standard normal distribution (with a mean of 0 and a variance of 1). Moreover, the generated random numbers are symmetrically distributed around 0 in a statistical sense, with a theoretical variance of 1, and the distribution shape is a typical bell-shaped curve. $Po_{RN}$ and $Po_{\varphi }$ represent two different random individuals in the population, and dim represents the dimension of the population. F is calculated by Equation (11), and F is uniformly distributed on [−1, 1].

(11) $$F=\left\{\begin{array}{l} 1\;\;\;if\;\left\lfloor 2\cdot ui+1\right\rfloor \;=\;1\\ -1\;\;\;\;if\;\left\lfloor 2\cdot ui+1\right\rfloor \;=\;2 \end{array}\right.$$

Here, ui is a random variable from 0 to 1 and it follows the same distribution pattern as uik. $\left\lfloor \cdot \right\rfloor$ represents the integer part, and ρ is obtained by Equation (12).

(12) $$\rho =\omega \cdot \left(1+sin\left(0.5\;\times \;loop\right)\right)$$

Here, $\sin\left(\cdot \right)$ represents the sine function, ω is a random variable with values ranging from 0 to 1, and the distribution patterns are all consistent with η. ε is obtained by Equation (13).

(13) $$\varepsilon =2\cdot fu\left(1,\dim\right)-1$$

Here, dim represents the dimension for solving the problem, $fu\left(1,\dim\right)$ indicates the number of randomly generated dim numbers from 1 to dim, and the distribution patterns of these numbers are consistent with those of uik.

If SF>1 is not satisfied, then it enters the exploitation stage. During this stage, the update of individuals is also randomly executed, which is specifically represented by Equation (14).

(14) $$Po_{inew}=\left\{\begin{array}{l} Po_{\mathrm{best}}+F\cdot \sqrt{\sum_{i=1}^{\dim}{\left(Po_{\mathrm{best}}-Po_{i}\right)}^{2}}\cdot \left(\sin\left(\tau \right)+\cos\left(\tau \right)\right)\;\;\;\mathrm{if}\;\nu <0.5\\ Po_{\mathrm{best}}+F\cdot G\cdot Levy\left(\dim\right)\cdot \left(Po_{\mathrm{best}}-Po_{i}\;\right)\;\;\;\;\;\;\mathrm{otherwise} \end{array}\right.$$

Here, ν represent a random variable with values ranging from 0 to 1, and its distribution pattern is the same as that of uik. $\cos\left(\cdot \right)$ represents the cosine function, and τ is calculated by Equation (15).

(15) $$\tau =\left(2\cdot \pi \cdot \xi \right)$$

where ξ represents a random number, the range is $\left[0,1\right]$, its distribution pattern is the same as that of ν, and G is obtained by Equation (16).

(16) $$G=2\cdot \left(1-\frac{\mathrm{loop}}{\mathrm{Maxloop}}\right)$$

$Levy\left(\dim\right)$ is calculated by Equation (17).

(17) $$Levy\left(\dim\right)=0.01\cdot \frac{\Gamma \left(2.5\right)\cdot \sin\left(0.75\cdot \pi \right)}{\Gamma \left(1.25\right)\cdot 1.5\cdot 2^{0.25}}\cdot \frac{ooi}{{\left|iio\right|}^{\frac{1}{1.5}}}$$

Here, $\Gamma \left(\cdot \right)$ represents the gamma function; ooi and iio, respectively, represent two random numbers with values ranging from 0 to 1 and the same distribution pattern as ξ.

### 4.2. Algorithm Implementation

The pseudo-code of the artificial lemming algorithm is shown in Algorithm 1, and the detailed procedure of the calculation method is as below:

Step 1: Initialize parameters, including population size, population dimension, the upper bound, and the lower bound of the problem.

Step 2: Initialize the population of the ALA according to the required parameters through Equation (8).

Step 3: Calculate the selection factor through Equation (9).

Step 4: Calculate the selection search direction factor through Equation (11).

Step 5: Update individual information through Equation (10) or Equation (14).

Step 6: Update the best individual.

Step 7: If loop<Maxloop, then return the optimal solution; otherwise, jump to Step 3.
**Algorithm 1**: Pseudo-code of ALA Input: Problem function: Fun, Solve the lower and upper bounds of the problem space: $\left[low,upp\right]$, Population size: N, Population dimension: dim, Total number of iterations: Maxloop
 Output: Optimal individual: $Po_{\mathrm{best}}$
**1:** Input: Fun, $\left[low,upp\right]$, N, dim, and Maxloop.**2:** Initialize the population through Equation (8).**3:** *while*  loop≤Maxloop *do***4:**   Calculate the selection factor SF using Equation (9).**5:**   Calculate the direction conversion factor F using Equation (11).**6:**   *if* SF>1 **7:**   Exploration stage**8:**        Update individuals through the exploration stage.**9:**   *else if* SF≤1 **10:**   Exploitation stage**11:**      Update individuals through the exploitation stage.**12:**   *end if***13:**   *if* $Fit_{inew}<Fit_{i}\;$$Po_{i}=Po_{inew}$**14:**   *end if***15:**   *if* $Fit_{inew}<Fit_{\mathrm{best}\;}$$Po_{\mathrm{best}}=Po_{inew}$**16:**   *end if***17:**   *end while***18:**   Output: $Po_{\mathrm{best}}$

## 5. The Improved ALA

When dealing with large-scale, complex, and variable optimization problems, the ALA struggles to perform effectively. The primary reasons include issues such as low population diversity and poor search capability in the original ALA. To address these limitations, this paper incorporates a collaborative second-order Bernstein two-dimensional chaotic initialization strategy into the standard ALA, aiming to enhance population dispersion. Additionally, a quadratic interpolation random mutation strategy is introduced in the exploitation phase to improve the algorithm’s exploitation capability, while an adaptive evolution strategy is integrated into the exploration phase to strengthen its exploration performance.

### 5.1. Initialization Strategy of Cooperative Bernstein Mixed Chaotic Function

**Figure 1:**
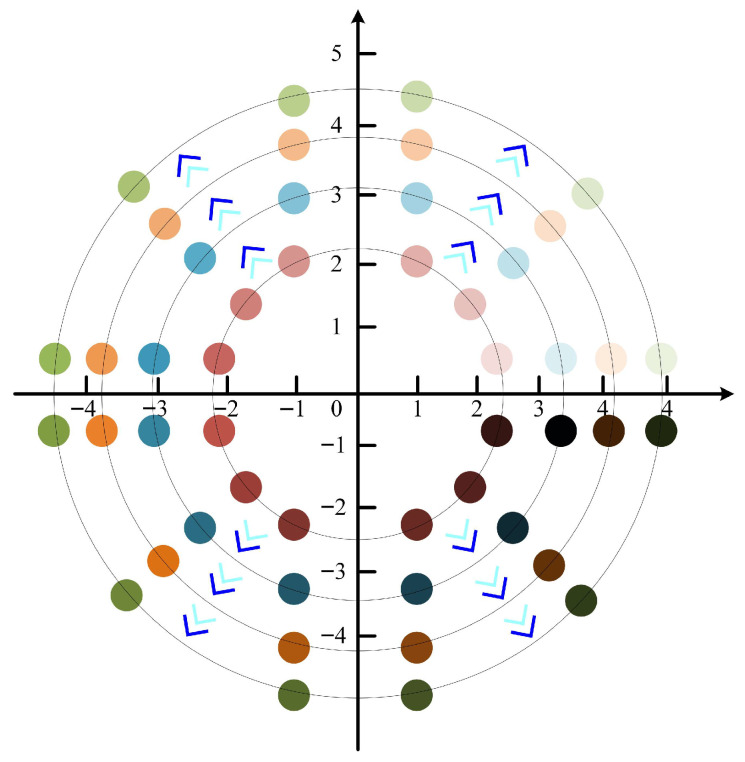
Perturbation process of the collaborative second-order chaotic Bernstein polynomial function.

Population dispersion is a core index in the optimization process of the algorithm, and its rationality directly affects the algorithm’s final convergence to the optimal solution. Based on this, this section proposes an initialization strategy of combining a Bernstein polynomial with a second-order chaotic function and embeds it in the framework of the ALA. The concrete logic of this strategy is as follows: Firstly, the position information of the initial population is disturbed by the second-order chaotic mapping, and the diversity of population distribution is enhanced by the ergodicity and randomness of chaotic behavior. Then, the deviation points generated in the disturbance process are adaptively corrected by the Bernstein polynomial to improve the dispersion of the initial population of the IALA and finally to realize its effective improvement in solving global optimization problems and curriculum-scheduling problems. It is worth noting that Bernstein polynomials have obvious advantages, such as concise expression and strong cross-domain applicability. The parameters determined based on it can not only ensure the numerical robustness, geometric shape-preserving, and order-upgrading flexibility of the basis function but also make the parameters to be estimated appear in the form of linearization, explicit, and constraints, which provides reliable support for the stable improvement of the algorithm’s performance. The specific implementation process is shown in Figure 1.

#### 5.1.1. Second-Order Chaotic Mapping Function

In the standard artificial lemming algorithm, the initial population positions are randomly generated within the upper and lower bounds of the problem space. This random generation mechanism, however, often leads to suboptimal spatial distribution characteristics—such as uneven dispersion or localized clustering—resulting in poor coverage of the feasible solution space. To address this limitation, this paper proposes to introduce perturbations to the initial positions of the population using a two-dimensional chaotic mapping function. Chaotic systems are characterized by sensitive dependence on initial conditions and ergodicity, which can effectively enhance the diversity and uniformity of the initial population distribution. By leveraging such a mapping, the population can explore a broader range of the solution space at the initial stage, thereby improving the overall richness of the population and laying a more robust foundation for subsequent evolutionary processes. The specific implementation is detailed in Equation (18).

(18) $$PoTd_{i}=\mathrm{mod}\left(PoC_{i}+0.2-\left(0.5/\left(2\cdot \pi \right)\right)\cdot \sin\left(2\cdot \pi \cdot Poc_{i}\right),1\right)$$

Here, $\mathrm{mod}\left(\cdot \right)$ represents the modulo division function. $PoC_{i}$ represents the i individual after being perturbed by the one-dimensional chaotic and is obtained by Equation (19).

(19) $$PoC_{i}=\cos\left(\frac{4}{\cos\left(Po_{i}\right)}\right)$$

Through the perturbation of high-order chaos, the individuals in the initial population of the IALA can explore a wider range of positions.

#### 5.1.2. Bernstein Polynomial

Bernstein polynomials have significant advantages such as approximation conformal properties, numerical stability, and deep correlation with Bezier curves. In the context of enhancing initial population richness for the algorithm, the coordination of the two-dimensional chaotic mapping function and the second-order Bernstein polynomial can achieve a synergistic effect. On one hand, the two-dimensional chaotic mapping, with its inherent ergodicity and randomness, ensures that the initial population can traverse the solution space more evenly, avoiding aggregation in local regions; on the other hand, the second-order Bernstein polynomial, with its smoothness and adjustable shape parameters, can further optimize the distribution of population positions by fine-tuning the local density of individuals, thereby making the initial population not only widely distributed but also rationally structured in density. This combination effectively addresses the potential defects of single mapping methods and significantly improves their richness. The specific form of the second-order Bernstein polynomial is given in Equation (20).

(20) $$\left\{\begin{array}{l} Bern_{1}={\left(1-ku\right)}^{2}\\ Bern_{2}=2\cdot ku\cdot \left(1-ku\right) \end{array}\right.$$

In Equation (20), $Bern_{1}$ represents the first-order Bernstein coefficient, $Bern_{2}$ represents the second-order Bernstein coefficient, ku represents a random variable between from 0 to 1, and its distribution pattern is consistent with that of uik.

**Figure 2:**
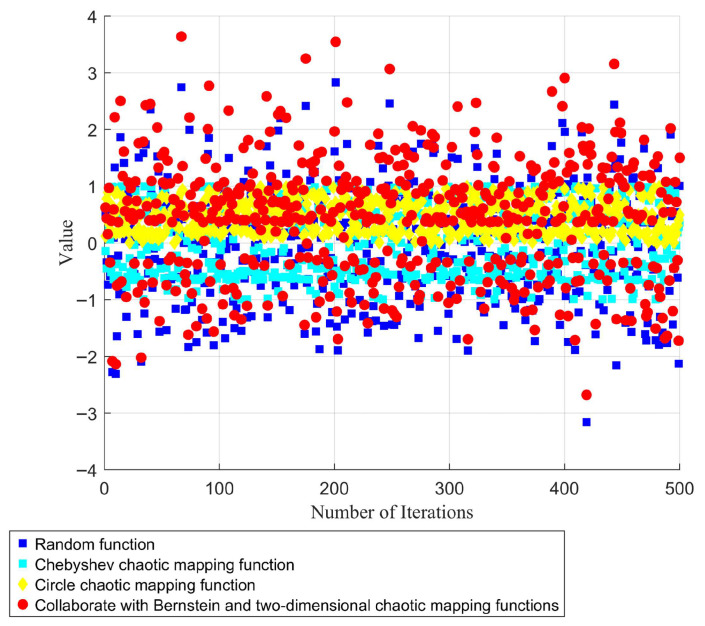
Comparison results of different initial strategies.

Ultimately, the initial population of individuals in the IALA is determined by Equation (21). As illustrated in Figure 2, individuals incorporating the second-order Bernstein polynomial and the two-dimensional chaotic mapping function are capable of covering a broader range within the solution space, while the initial population exhibits the highest degree of dispersion. This enhanced distribution not only expands the coverage of potential solutions but also ensures a more uniform spread across the solution domain, laying a robust foundation for subsequent iterative optimization processes.

(21) $$IPo_{i}=Bern_{1}\cdot Po_{i}+Bern_{2}\cdot PoTd_{i}$$

In Equation (21), $IPo_{i}$ is the i individual of the IALA.

### 5.2. Quadratic Interpolation Random Variation

**Figure 3:**
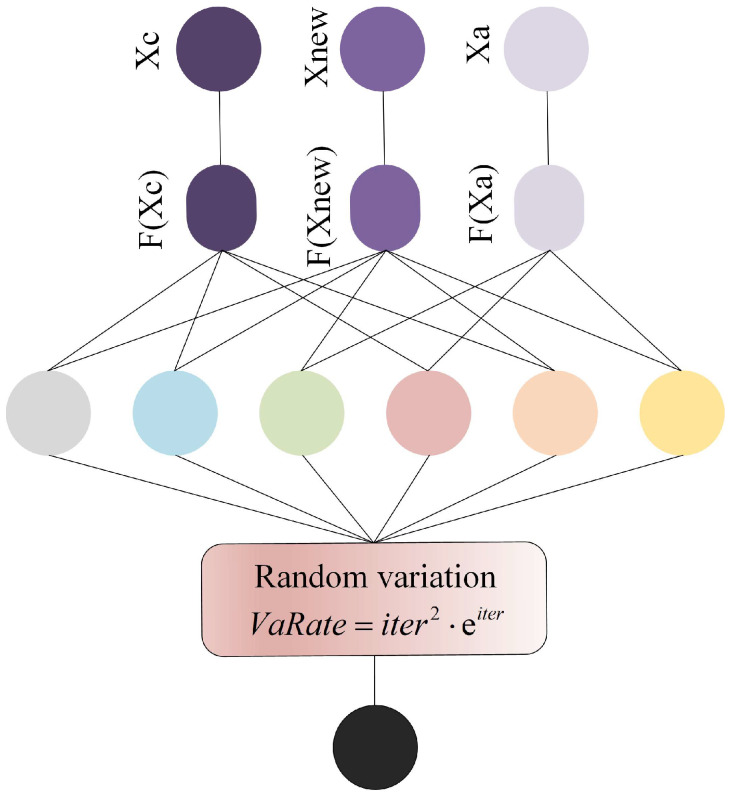
Quadratic interpolation random mutation process.

When the ALA is applied to solve cloud task-scheduling problems, a notable issue arises: the system tends to exhibit relatively long response times. This inefficiency can be primarily attributed to the insufficient development capability of the ALA during the optimization process. Specifically, the algorithm’s weak ability to explore and exploit fine-grained regions within the solution space limits its capacity to accurately converge to the global optimal solution. As a result, it fails to identify the most optimal scheduling strategy with sufficient precision—often lingering in suboptimal regions, which in turn prolongs the overall task-processing cycle. To address this limitation, this section introduces a quadratic interpolation random mutation strategy, aiming to enhance the ALA’s utilization capability of potential solution spaces. The quadratic interpolation component leverages the information of existing individuals to construct a smooth local approximation model, enabling more targeted exploration around promising regions; meanwhile, the random mutation mechanism introduces controlled diversity, preventing the algorithm from prematurely stagnating in local optima. Together, these two components allow the mutant factors to better balance the positions of individuals; they not only refine the search in promising areas (strengthening development) but also maintain a certain degree of exploration in untapped regions. The effectiveness of this balance is visually illustrated in Figure 3, which contrasts the distribution of individuals before and after the introduction of the strategy. Additionally, the specific mathematical process of how individuals update their positions under this strategy is detailed in Equation (22).

(22) $$QIIPo_{i}=\frac{f\left(IPo_{r1}\right)\cdot UPE1+f\left(IPo_{inew}\right)\cdot UPE2+f\left(IPo_{r2}\right)\cdot UPE3}{f\left(IPo_{r1}\right)\cdot LOE1+f\left(IPo_{inew}\right)\cdot LOE2+f\left(IPo_{r2}\right)\cdot LOE3}$$

In Equation (22), $IPo_{inew}$ represents the individual updated from Equation (10), and $IPo_{r1}$ and $IPo_{r2}$, respectively, represent two distinct individuals that are not $IPo_{inew}$. $f\left(IPo_{r1}\right)$, $f\left(IPo_{inew}\right)$, and $f\left(IPo_{r2}\right)$, respectively, represent the fitness values of individuals $IPo_{r1}$, $IPo_{inew}$, and $IPo_{r2}$. UPE1, UPE2, and UPE3 are calculated by Equation (23).

(23) $$\left\{\begin{array}{l} UPE1=IPo_{inew}^{2}-IPo_{r2}^{2}\\ UPE2=IPo_{r2}^{2}-IPo_{r1}^{2}\\ UPE3=IPo_{r1}^{2}-IPo_{inew}^{2} \end{array}\right.$$

LOE1, LOE2, and LOE3 are calculated by Equation (24).

(24) $$\left\{\begin{array}{l} LOE1=IPo_{inew}-IPo_{r2}\\ LOE2=IPo_{r2}-IPo_{r1}\\ LOE3=IPo_{r1}-IPo_{inew} \end{array}\right.$$

Then, individual variation is controlled through the mutation rate VaR, which is calculated by Equation (25).

(25) $$VaR=1-\frac{1}{{\mathrm{loop}}^{2}\cdot e^{\mathrm{loop}}}$$

Here, $e^{\left(\cdot \right)}$ represents an exponential function with base e. The final quadratic interpolation random variation is shown in Equation (26).

(26) $$QIIRVIPo_{i}=VaR\cdot QIIPo_{i}$$

Throughout the iterative process, by employing the quadratic interpolation random mutation strategy, the population can sustain a high degree of diversity. This not only prevents premature convergence caused by the loss of population variability but also effectively bolsters the algorithm’s exploitation ability—enabling it to delve deeper into promising solution regions while maintaining the breadth of exploration.

### 5.3. Adaptive Evolutionary Strategy

The traditional ALA is prone to premature convergence when tackling global optimization problems, often resulting in the algorithm converging to a local extremum rather than the global optimal solution. This phenomenon can be primarily attributed to the algorithm’s insufficient exploration capability during the iterative process; as the optimization proceeds, individuals tend to fall into stagnation, lacking the ability to effectively traverse unvisited regions of the solution space. Such a limitation not only restricts the algorithm from escaping local optima but also leads to noticeable deficiencies in terms of stability—especially when solving complex problems like cloud task scheduling or other large-scale global optimization tasks. For instance, in cloud task-scheduling scenarios, this might manifest as suboptimal resource allocation, prolonged task completion times, or inconsistent performance across different scheduling instances.

**Figure 4:**
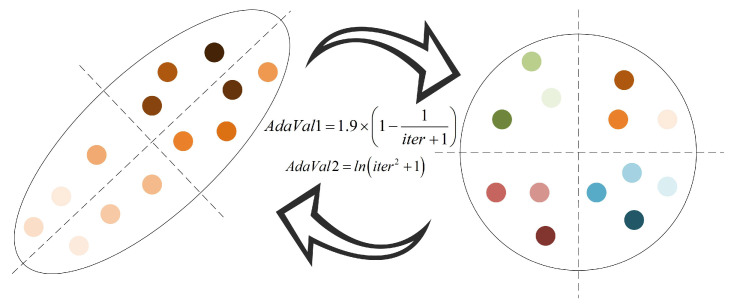
Adaptive evolutionary process.

To address these issues, this paper introduces an adaptive evolution strategy designed to enhance the algorithm’s exploration performance. This strategy dynamically adjusts the migration behavior of individuals during the exploration phase, enabling them to migrate across a broader range of the solution space. By adaptively tuning parameters such as migration step size and direction based on the current population distribution, the strategy effectively prevents individuals from clustering in local regions and promotes more extensive coverage of potential optimal solutions. Consequently, this improvement not only mitigates the risk of premature convergence but also enhances the algorithm’s overall robustness and optimization accuracy. The evolutionary process of individuals under this strategy is visualized in Figure 4, and the specific mathematical formulation governing individual evolution is provided in Equation (27).

(27) $$AEIPo_{i}=IPo_{inew}-AdVa1\cdot IPo_{\mathrm{best}}+AdVa2\cdot ff\left(low,upp\right)$$

In Equation (27), $ff\left(\cdot \right)$ generates a random number, and $ff\left(low,upp\right)$ represents randomly generating an integer between the higher and lower limits. AdVa1 and AdVa2, respectively, represent the adaptive evolution parameters and are calculated by Equation (28).

(28) $$\left\{\begin{array}{l} AdVa1=1.9\cdot \left(1-\frac{1}{\mathrm{loop}+1}\right)\\ AdVa2=\ln\left({\mathrm{loop}}^{2}+1\right) \end{array}\right.$$

Here, individuals will gradually converge to the current optimal individuals in the iterative process. Therefore, adaptive parameters are introduced into the proposed strategy, the core purpose of which is to dynamically adjust the individual position. Because the process of exploration and exploitation needs to be balanced through the change of parameters, the value of this parameter needs to increase gradually with the iterative process, so its formula is set as a linear non-decreasing function.

Through adaptive parameter correction, the algorithm can empower each individual to traverse a more extensive solution space during the exploration stage. On one hand, by guiding individuals to move away from the current optimal solution—with parameters dynamically adjusted based on iterative progress—it effectively enhances their ability to break free from the confinement of local optima, preventing the algorithm from falling into premature stagnation. On the other hand, leveraging the adaptive search mechanism that drives individuals to actively explore new positions in the solution space, the algorithm ensures that even in the later stages of iteration, when the population might otherwise tend to converge, it can still maintain a high level of population dispersion and sustained exploration dynamics. This dual effect not only expands the algorithm’s coverage of potential optimal regions but also preserves its capability to discover superior solutions, thereby laying a solid foundation for improving overall optimization performance.

### 5.4. The Implementation of the Improved Artificial Lemming Algorithm

**Figure 5:**
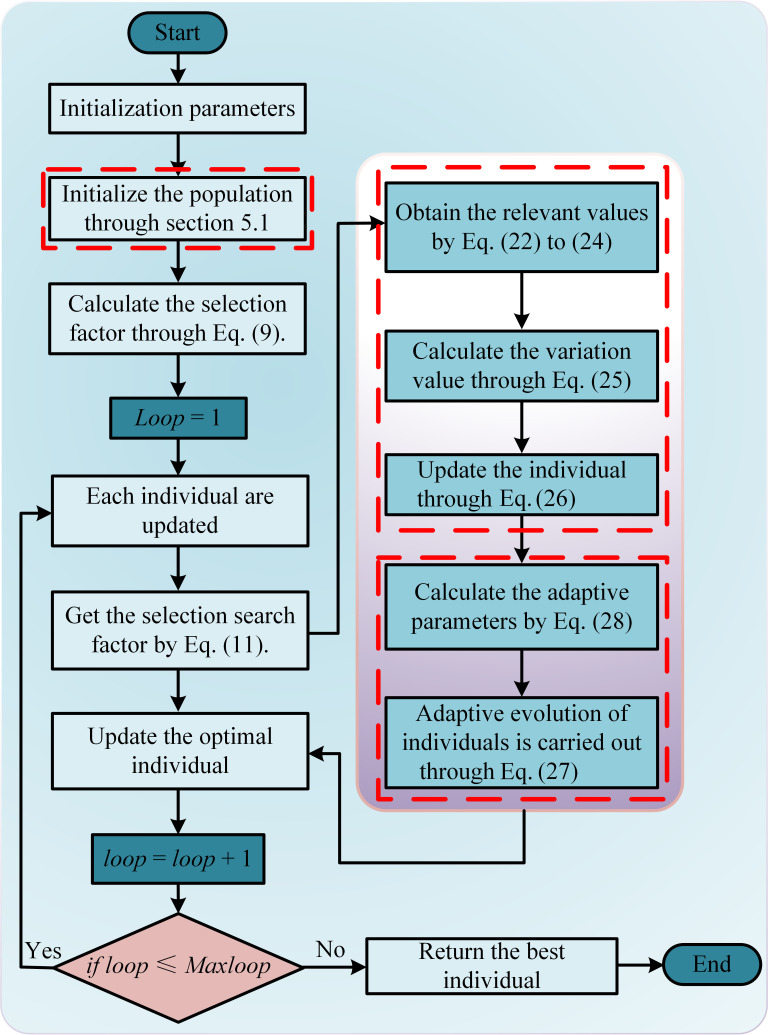
The specific process of IALA.

Algorithm 2 provides the pseudo-code of the IALA. Figure 5 shows the algorithm flow of the IALA, and its specific implementation is described as follows.

Step 1: Initialize the required parameters, including population size, population dimension, the upper bound, and the lower bound of the problem space.

Step 2: Initialize the population according to the parameters by Equation (18).

Step 3: Calculate the selection factor through Equation (9).

Step 4: Calculate the selection search direction factor through Equation (11).

Step 5: Perform the corresponding stage based on the selection factor value. If it is SF>1, then proceed to Step 6; otherwise, proceed to Step 8.

Step 6: Update individuals during the exploitation phase.

Step 7: Update individuals through a quadratic interpolation random mutation method.

Step 8: Update individuals through the exploration stage.

Step 9: Update individuals through adaptive strategies.

Step 10: Update the best and worst individuals.

Step 11: If loop<Maxloop, then return the optimal solution; otherwise, jump to Step 3.
**Algorithm 2:** Pseudo-code of IALA Input: Problem function: Fun, The lower and upper bounds of the problem: $\left[low,upp\right]$, Population size: N, Population dimension: dim, Total number of iterations: Maxloop
 Output: Optimal individual: $IPo_{\mathrm{best}}$
**1:** Input: Fun, $\left[low,upp\right]$, N, dim, and Maxloop.**2:** Initialize the population of IALA according to Equation (28).**3:** *while* loop≤Maxloop *do***4:**   Calculate the selection factor SF using Equation (9).**5:**   Calculate the direction conversion factor F using Equation (11).**6:**   *if*  SF>1 **7:**   Exploration stage**8:**     Update individuals through the exploitation stage.**9:**     $QIIRVIPo_{i}$ is obtained by Equation (26).**10:**     Make a greedy choice of an individual $QIIRVIPo_{i}$.**11:**     *else if* SF≤1 **12:**     Exploitation stage**13:**       Update individuals through the exploration stage.**14:**       $AEIPo_{i}$ is obtained by Equation (27).**15:**       Make a greedy choice of an individual $AEIPo_{i}$.**16:**     *end if***17:**     *if* $Fit_{inew}<Fit_{i}\;$$IPo_{i}=IPo_{inew}$**18:**     *end if***19:**     *if* $Fit_{inew}<Fit_{\mathrm{best}\;}$$IPo_{\mathrm{best}}=IPo_{inew}$**20:**     *end if***21:**   *end while***22:**   Output: $IPo_{\mathrm{best}}$

## 6. Experimental Analysis

To verify the effectiveness of the proposed algorithm, this section first conducts experiments on the above three strategies, systematically verifying and fully demonstrating the significant advantages of the proposed strategy. Secondly, to highlight the unique advantages of the IALA in solving global optimization problems, it is applied together with the classical algorithm and the improved algorithm to the CEC2021 test problem for comparative analysis. Finally, the performance of the IALA was verified again through cloud scheduling experiments, further enhancing its practicality and reliability. All experiments were completed under the Windows 11 operating system, the compilation environment was MATLAB 2024b, and the hardware platform was equipped with a 12th Gen Intel (R) Core (TM) i5-12400 processor (with a main frequency of 2.50 GHz).

### 6.1. Strategy Effectiveness Analysis

In order to verify the effectiveness of the proposed strategy, this section carries out systematic verification. The CEC2021 test function is selected as the verification carrier, mainly based on its two advantages: First, the test function has strong novelty and is widely used to evaluate the ability of the algorithm to deal with global optimization problems. Second, it covers unimodal, multimodal, complex, and mixed problems and can comprehensively test the performance of the strategy in different scenarios. The specific verification process is as follows: Firstly, the effectiveness of the proposed initialization strategy is evaluated by the population dispersion index. Furthermore, the algorithm is applied to the CEC2021 test problem together with two other comparison strategies, and the overall efficiency of the proposed strategy is further verified by horizontal comparison.

#### 6.1.1. Validation of the Proposed Initialization Strategy

To verify the effectiveness of the cooperative Bernstein polynomial and second-order chaotic mapping initialization strategy, the standard ALA and ALA with only the above strategy are verified by the CEC2021 problem, and the ALA with only the above strategy is called the IALA.

To verify the practicability of the strategy, population dispersion is introduced to measure the distribution of individuals. If the population dispersion is greater, then the individual positions are more dispersed, and the algorithm is more likely to obtain a more accurate optimal solution. Population dispersion $\left(PDD\right)$ is expressed by Equation (29):

(29) $$PDD\left(\mathrm{loop}\right)=\sqrt{\sum_{i=1}^{N}\sum_{j=1}^{\dim}{\left(IPo_{i}^{j}-CV\left(\mathrm{loop}\right)\right)}^{2}}$$

in Equation (29), the value of population dispersion of $PDD\left(\mathrm{loop}\right)$ at the loop iteration. $IPo_{i}^{j}$ represents the information of the ith individual in the jth dimension, and $CV\left(\mathrm{loop}\right)$ represents the value of the population centroid in the loop iteration, which is calculated by Equation (30).

(30) $$CV\left(\mathrm{loop}\right)=\frac{1}{\dim}\cdot \sum_{I=1}^{N}IPo_{i}^{j}$$

**Figure 6:**
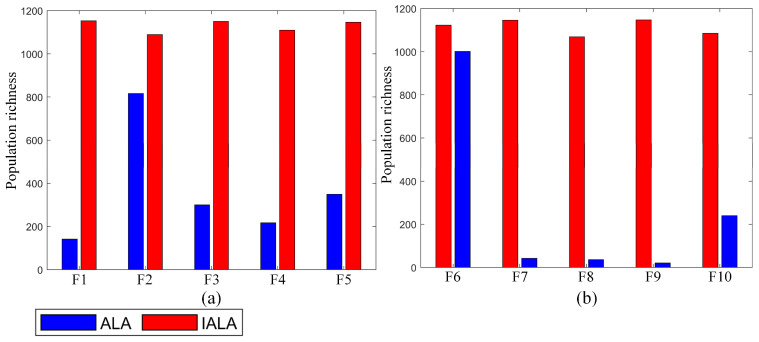
Population diversity results of ALA and IALA on CEC2021((**a**) the result of the CEC2021 F1–F5; (**b**) the result of the CEC2021 F6–F10).

In order to fully highlight the effectiveness of the proposed strategy, the experiment set the initial population number to 20, the population dimension to 20, and the total number of iterations to 500. See Table 1 for the relevant parameters of CEC2021. In the CEC2021 test problem, only the dispersion of the initial population is calculated, and the experimental results under the above conditions are shown in Figure 6. In Figure 6, the blue column represents the population dispersion value of the original ALA on the CEC2021 test problem, while the red column corresponds to the population dispersion value of ILA on the same test problem, only with the introduction of the cooperative Bernstein polynomial and the second-order chaotic mapping initialization strategy. By observing Figure 6, it can be found that the population dispersion of the IALA is significantly higher than that of the original ALA in the test function range from F1 to F10, which strongly proves the validity of the put-forward tactic. As is evident from Figure 6a, when handling single-peak straightforward problems, although the initial population dispersion of the ALA is above 100, the initial population dispersion of the IALA is above 1000. Moreover, the initial population dispersion values of ILA are higher than those of the ALA on the corresponding test problems, which shows that ILA has the potential to explore more accurate optimal solutions when handling straightforward issues. As shown in Figure 6b that when dealing with mixed complex problems, the ALA shows poor initialization performance, especially on the test functions F7, F8, and F9; its initial population dispersion values are all below 100. Although the test function F10 has been improved, on the other hand, the IALA maintains a high initial population dispersion from test questions F6 to F10. Generally speaking, on the CEC2021 test, the average dispersion of the initial population of the IALA is about 3.4 times that of the initial population of the ALA.

#### 6.1.2. Verification of the Effectiveness of the Proposed Assistance Strategy

To verify the improvement effect of the proposed quadratic interpolation random mutation on the algorithm development capability and the auxiliary role of the adaptive evolution strategy in the exploration stage, this section conducts verification by applying it to the CEC2021 function problem. Specifically, the ALA that integrates only the quadratic interpolation random mutation strategy is named the quadratic interpolation random mutation strategy-based ALA (QIRMALA). The ALA that only integrates the adaptive evolutionary strategy is named the adaptive evolutionary strategy-based ALA (AEALA). The ALA that integrates both of the above strategies simultaneously is named the QIAEALA.

The experiment set the population size to 50, the population dimension to 10, and the total number of iterations to 500. The remaining relevant parameters are shown in Table 1, and the experimental results are presented in Table 2. As can be seen from the data in Table 2, the performance of the QIRMALA, AEALA, and QIAEALA in the CEC2021 test problems is all superior to that of the traditional ALA, which fully demonstrates that the proposed strategy has a significant improvement effect on the standard ALA. Specifically, compared with the original ALA, the performance improvement of the QIRMALA on the test functions F1–F4 is particularly significant, confirming the effectiveness of the quadratic interpolation random mutation strategy. However, in the complex and variable F5–F10 test functions, although the optimization effect has improved, it is not significant enough. Complementary to this, the AEALA almost always demonstrated excellent performance in the test problems where the QIRMALA performed poorly, indicating that the introduction of an adaptive evolution strategy can effectively enhance the ALA’s ability to solve complex problems. The QIAEALA, which integrates the two strategies, performed exceptionally well overall on the CEC2021 test question set and ranked first in the average ranking. This result fully demonstrates that after integrating the two strategies in a coordinated manner into the ALA, the comprehensive performance of the standard algorithm can be significantly enhanced, making it more capable of handling complex and variable global optimization problems.

### 6.2. Global Optimization Ability Test

To effectively verify the IALA’s problem-solving ability in global optimization problems, this subsection will discuss the IALA with Ant Colony Optimization (ACO) [50], Differential Evolution (DE) [51], and Particle Swarm Optimization (PSO) [52], Hiking Optimization Algorithm (HOA) [53], Whale Migration Algorithm (WMA) [54] and Red-billed Blue Magpie Optimizer (RBMO) [55] was jointly applied to the CEC2021 test problem for comparative verification, and the parameter Settings of the other algorithms were consistent with those in the references.

**Figure 7:**
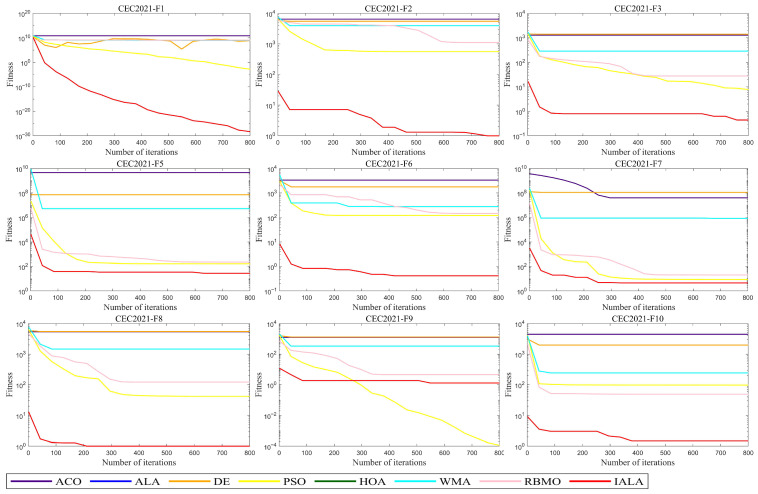
The test results of IALA and other algorithms at CEC2021.

**Figure 8:**
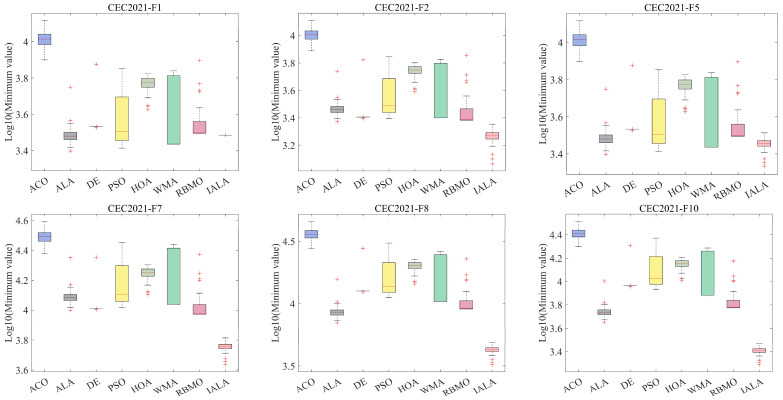
The test results of IALA and other algorithms on the box diagram at CEC2021.

The experimental environment remains consistent with the above. In this section, the population size is set to 50, the problem dimension to 20, and the maximum number of iterations to 800. Tests were conducted on the IALA and other comparative algorithms, with the experimental results presented in Figure 7 and Figure 8.

As evident from Figure 7, across almost all test problems, the initial value of the IALA is lower than those of other algorithms, which indicates that the validation of the proposed initialization strategy has exerted a substantial impact. This enables the IALA to achieve higher accuracy in optimal solutions even at the first iteration compared to other algorithms. Further analysis shows that when handling simple test functions F1–F3, the standard ALA almost falls into local optima throughout the entire iteration process, whereas the IALA successfully avoids this predicament from the very start. For complex problems F5–F7, the IALA still maintains favorable performance, particularly demonstrating a cliff-like leading advantage in the F6 problem. Although the IALA did not reach the optimal value on the F9 test function, it still ranks second. Overall, the IALA exhibits excellent performance in addressing global optimization problems, which further validates the effectiveness of the proposed auxiliary strategy.

To verify the stability of the proposed IALA, the algorithm and other comparative methods were further employed to solve CEC2021 test problems, with the experimental parameters remaining unchanged. Each algorithm was executed independently 50 times, and the results were analyzed, with the boxplot results shown in Figure 8. Across various types of problems in CEC2021, the IALA demonstrates a cliff-like leading performance, which fully confirms that its stability is superior to that of other algorithms. Specifically, in simple problems, although the IALA does not outperform others by a large margin, it still maintains the top position. However, when dealing with complex and variable combinatorial problems, the performance of other algorithms deteriorates significantly, whereas the IALA can still retain good stability, confirming that it is a highly robust algorithm.

### 6.3. Cloud Task-Scheduling Problem Test

To further verify the practical impact and application value of the proposed IALA when tackling cloud task-scheduling problems, this subsection selects cloud task scheduling—a representative and complex scenario—as the validation platform and conducts a systematic assessment of the algorithm’s effectiveness. Taking into account the dynamic variation of task scales in cloud environments, both small-scale and large-scale sets of task-scheduling simulation experiments are designed. From multiple dimensions—load cost, time cost, price cost, and others—the performance of the IALA is comprehensively evaluated under different scenarios, thereby clarifying its applicable scope and optimization potential in cloud task-scheduling problems.

#### 6.3.1. Analysis of Small-Scale Cloud Task Scheduling

**Figure 9:**
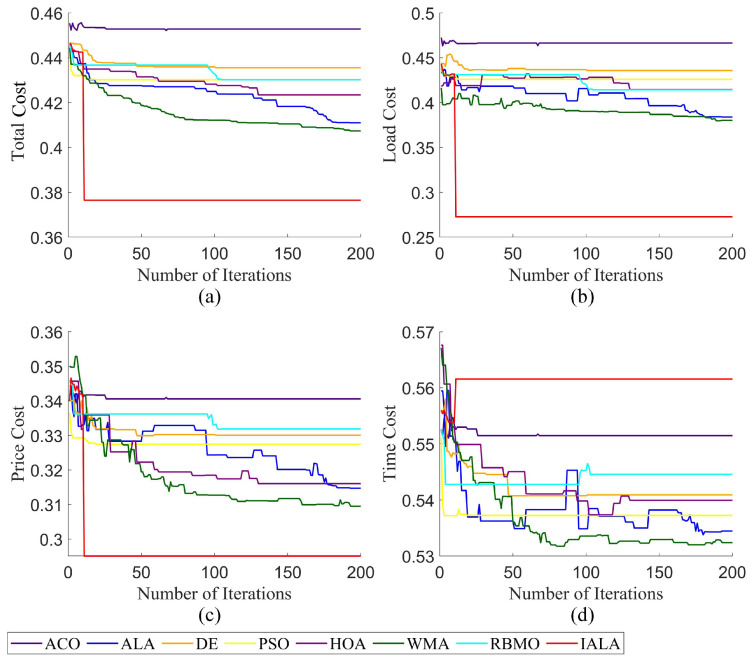
The scheduling result of small-batch tasks ((**a**) total cost result; (**b**) load cost result; (**c**) price cost result; (**d**) time cost result).

To verify the solving ability of the IALA in practical engineering problems, this section conducts comparative experiments by applying the IALA together with the ACO, ALA, DE, PSO, HOA, WMA, and RBMO to the small-batch cloud task-scheduling problem. The experimental parameters are set as follows: 150 tasks, 40 virtual machines, a group scale of 40, and a total of 200 repetitions. The remaining relevant parameters are detailed in Table 3, and the laboratory findings are displayed in Figure 9.

Laboratory findings presented in Figure 9 demonstrate that when solving small-scale cloud task-scheduling problems, the IALA exhibits significantly superior comprehensive performance compared to both traditional classical algorithms (e.g., ACO, DE, PSO) and emerging intelligent algorithms (e.g., HOA, WMA, RBMO). A detailed analysis is as follows: In the time cost dimension (Figure 9a), the IALA achieves a notable reduction of approximately 8% compared to the baseline ALA algorithm. This data underscores that even in practical engineering scheduling scenarios, the IALA maintains stable and exceptional optimization capabilities, reflecting targeted enhancements to core performance metrics. The IALA’s advantages become more pronounced in optimizing load overhead and capital costs: Chart comparisons intuitively show that its performance in load cost control far surpasses the ALA, enabling more efficient balancing of virtual machine resource loads and preventing local overload or resource idleness. In capital cost optimization, the IALA also demonstrates a clear improvement trend.

While the IALA’s time cost optimization extent has not yet reached the ideal target, its overall performance remains leading when comprehensively evaluating the three core metrics of time, load, and capital. It follows that the IALA is a robust optimization algorithm suitable for small-scale cloud task-scheduling scenarios, holding significant practical value in engineering applications.

#### 6.3.2. Analysis of Large-Scale Cloud Task Scheduling

**Figure 10:**
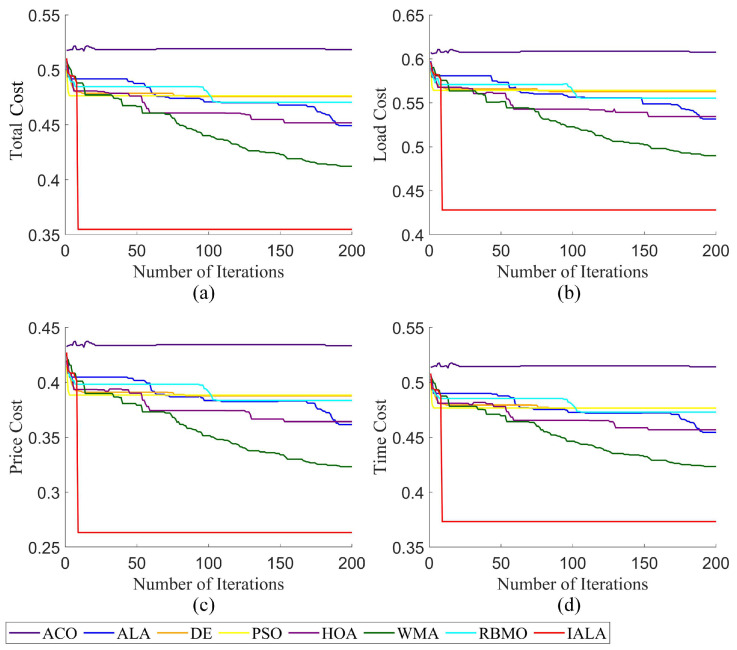
Large-scale task-scheduling results ((**a**) total cost result; (**b**) load cost result; (**c**) price cost result; (**d**) time cost result).

To verify the universality of the IALA in cloud task-scheduling problems, this section further adjusts the experimental scale: the number of tasks is set to 400, the number of virtual machines is increased to 70, while the population size and the number of iterations remain consistent with those in Section 6.3.2. Other relevant parameters are detailed in Table 4. Based on the above Settings, all algorithms were re-verified, and the experimental results are shown in Figure 10. Experimental results in Figure 10a show that when the task scale is expanded to 400, the IALA still maintains excellent optimization capabilities during scheduling, fully demonstrating its stability in complex scenarios. In contrast, the baseline ALA exhibits notable shortcomings in large-scale task scheduling: its performance lags in load balancing, time cost reduction, and capital cost optimization, failing to meet the requirements of large-scale scheduling scenarios. Specifically, the IALA’s optimization effects are particularly prominent: in terms of load overhead, it achieves a reduction of approximately 19% compared to the ALA, effectively alleviating resource load pressure on virtual machine clusters and reducing the risk of task delays caused by local overload. In capital cost optimization, the IALA’s improvement rate reaches around 28%, significantly lowering overall scheduling costs through more precise resource matching strategies. Notably, even in the time cost indicator—where optimization room was previously limited—the IALA shows breakthrough progress, further confirming its ability to enhance synergy across multi-dimensional optimization objectives. Based on experimental data from large-scale scenarios, the IALA not only performs well in small-scale task scheduling but also maintains strong optimization performance in complex scenarios where both task quantity and virtual machine scale increase simultaneously.

## 7. Summary and Prospect

In the research work of this paper, the basic ALA is mainly improved through three key strategies. Firstly, the initialization strategy combining the collaborative second-order Bernstein polynomial with the two-dimensional chaotic mapping function is introduced into the ALA. This improvement significantly enhances the dispersion of the initial population, lays a better basis for the following exploration procedure of the algorithm, and thereby effectively improves the precision of finally locating the optimal solution. Secondly, the quadratic interpolation random variation strategy is introduced in the algorithm development stage. This strategy can assist individuals in the population to maintain their search advantages while approaching the local optimal solution, avoiding the loss of diversity due to excessive convergence. Secondly, in the exploration stage, an adaptive evolution strategy is introduced. By dynamically adjusting the exploration behavior of individuals, their exploration ability in the global space is enhanced, ensuring that the algorithm can still maintain a high population richness in the later stage of iteration, thereby reducing the risk of falling into a local optimum. To verify the performance of the improved algorithm, this paper applies the proposed IALA to the CEC2021 testing function collection and cloud task-scheduling issues of varying sizes, respectively. The laboratory findings indicate that the IALA demonstrates efficient solution capabilities in both global optimization problems and task-scheduling scenarios, fully verifying its effectiveness and practicability.

However, although the IALA has demonstrated excellent performance in the above-mentioned tests and applications, it is still a single-objective optimization algorithm at present and is difficult to directly meet the requirements of multi-objective optimization scenarios. Therefore, future research work will first focus on the expansion of multi-objective optimization capabilities, exploring how to enable the IALA to simultaneously optimize multiple conflicting objectives through strategic improvements. In addition, some parameter settings in the IALA are currently fixed, which may affect their adaptability to different problem scenarios. In the future, we will also consider introducing parameter adaptive adjustment strategies or intelligent optimization mechanisms to address this issue, further enhancing the universality and scene adaptability of the algorithm. In addition, for the proposed initialization strategy, chaotic mapping is coordinated, but chaotic mapping still has uncertainties. Therefore, future work can be carried out based on this.

## Figures and Tables

**Table 1:** Information related to CEC2021.

Index	Types	Name	Optimum
F1	Unimodal function	Shifted and Rotated Bent Cigar Function (CEC 2017 F1)	100
F2	Basic functions	Shifted and Rotated Schwefel’s Function (CEC 2014 F11)	1100
F3		Shifted and Rotated Lunacek bi-Rastrigin Function (CEC 2017 F7)	700
F4		Expanded Rosenbrock’s plus Griewangk’s Function (CEC 2017 F_19_)	1900
F5	Hybrid functions	Hybrid Function 1 (N=3) (CEC 2014 F17)	1700
F6		Hybrid Function 2 (N=4) (CEC 2017 F16)	1600
F7		Hybrid Function 3 (N=5) (CEC 2014 F21)	2100
F8	Composition functions	Composition Function 1 (N=3) (CEC 2017 F22)	2200
F9		Composition Function 2 (N=4) (CEC 2017 F24)	2400
F10		Composition Function 3 (N=5) (CEC 2017 F25)	2500
Search range: [−100, 100]			

**Table 2:** Test results of different strategies on the CEC2021 test function.

Test Functions	ALA	QIRMALA	AEALA	QIAEALA
CEC2021-F1	3.1179 × 10^−12^	6.78 × 10^−13^	7.71 × 10^−14^	2.1 × 10^−16^
CEC2021-F2	2.35 × 10^−3^	1.17 × 10^−4^	2.89 × 10^−6^	3.13 × 10^−7^
CEC2021-F3	1.6066 × 10^−17^	2.341 × 10^−18^	1.892 × 10^−18^	3.341 × 10^−20^
CEC2021-F4	1.1102 × 10^−16^	2.134 × 10^−17^	2.897 × 10^−18^	2.21 × 10^−20^
CEC2021-F5	8.03 × 10^−4^	1.189 × 10^−4^	1.652 × 10^−5^	2.218 × 10^−7^
CEC2021-F6	1.01	2.213 × 10^−1^	9.675 × 10^−2^	3.341 × 10^−4^
CEC2021-F7	5.16 × 10^−1^	6.12 × 10^−2^	7.365 × 10^−3^	5.572 × 10^−5^
CEC2021-F8	8.1182 × 10^−5^	6.172 × 10^−6^	6.234 × 10^−6^	5.523 × 10^−7^
CEC2021-F9	2.0505 × 10^−6^	1.892 × 10^−8^	2.256 × 10^−7^	3.4512 × 10^−9^
CEC2021-F10	1.42 × 10^−1^	3.32 × 10^−2^	4.324 × 10^−2^	7.982 × 10^−4^
Average ranking	4	2.7	2.2	1

**Table 3:** Resource parameters related to small-scale task scheduling.

Parameters	Range (VMs)	Range (Tasks)
Ca	[400, 600]	[20, 60]
Lo	[200, 600]	[60, 110]
RB	[200, 350]	[40, 70]

**Table 4:** Parameters related to large-scale task scheduling.

Parameters	Range (VMs)	Range (Tasks)
Ca	[1530, 2860]	[300, 800]
Lo	[2148, 4196]	[100, 150]
RB	[600, 800]	[170, 200]

## Data Availability

All the data in this article can be obtained by contacting the corresponding author.
